# The Role of Book Features in Young Children's Transfer of Information from Picture Books to Real-World Contexts

**DOI:** 10.3389/fpsyg.2018.00050

**Published:** 2018-02-06

**Authors:** Gabrielle A. Strouse, Angela Nyhout, Patricia A. Ganea

**Affiliations:** ^1^Counselling and Psychology in Education, University of South Dakota, Vermillion, SD, United States; ^2^Department of Applied Psychology and Human Development, University of Toronto, Toronto, ON, Canada

**Keywords:** picture books, symbolic development, analogical reasoning, fantasy distinction, learning, transfer

## Abstract

Picture books are an important source of new language, concepts, and lessons for young children. A large body of research has documented the nature of parent-child interactions during shared book reading. A new body of research has begun to investigate the features of picture books that support children's learning and transfer of that information to the real world. In this paper, we discuss how children's symbolic development, analogical reasoning, and reasoning about fantasy may constrain their ability to take away content information from picture books. We then review the nascent body of findings that has focused on the impact of picture book features on children's learning and transfer of words and letters, science concepts, problem solutions, and morals from picture books. In each domain of learning we discuss how children's development may interact with book features to impact their learning. We conclude that children's ability to learn and transfer content from picture books can be disrupted by some book features and research should directly examine the interaction between children's developing abilities and book characteristics on children's learning.

On the bookshelf of a pre-reader, one may find storybooks that take children to magical worlds with fantastical characters, to faraway lands with unique animals and customs, or keep them close to home with tales about backyard bullies or trips to the dentist. Alongside these, one may also find factual books about outer space, underwater creatures, or pre-historic dinosaurs. These books may differ from one another in a number of their features, including their genre, presence of fantastical elements, pictorial realism, and use of factual language. Children are expected to learn facts, concepts, or values and apply them to real life. The current body of evidence on whether children can learn and transfer new content from picture books suggests that it is important to consider both the dimensions on which the books vary and children's developing abilities. In this review we summarize the existing evidence on the effect of book features on young children's learning and transfer and outline three developmental abilities that may interact with whether children's learning will be impacted by the presence or absence of those book features.

The majority of past research on picture books has focused on the nature of the book sharing interaction between adults and children (e.g., Fletcher and Reese, 2005). This large body of research demonstrates that different picture book features shape the interactions that take place between dyads; for example expository texts lead to more maternal teaching during reading than narrative texts (Pellegrini et al., 1990), less specific language (Nyhout and O'Neill, 2014), and more maternal feedback (Moschovaki and Meadows, 2005), whereas high quality illustrations lead to more child labeling of pictures (Potter and Haynes, 2000). Mothers are more likely to point and label letters for their young children when interacting with a plain book than a book with manipulative features and children also vocalize most often about the letters and pictures in the plain book (Chiong and DeLoache, 2012). Thus, aspects of the book can alter what both parents and children focus on. Recently the impact of book features directly on children's learning from print picture books has also received increasing attention in developmental research. Two recent reviews have provided targeted overviews of features that support vocabulary learning (Wasik et al., 2016) and learning from fictional media more broadly (Hopkins and Weisberg, 2017). These reviews indicate that children are selective in their learning and that properties of media can affect children's learning. In the current review, we focus specifically on learning from picture books, with the goal of outlining how three key developmental factors (symbolic development, analogical reasoning, and reasoning about fantasy) may influence young children's learning and transfer from books that vary across various dimensions. We will focus on domains of learning where most of the research on picture book features so far has been conducted with pre-readers: learning of words and letters, science concepts, problem solutions, and morals.

One goal of educational book-sharing interactions is for children to build generalizable knowledge they can learn and transfer outside of storybooks to everyday situations. By *learning*, we refer to the child's ability to recognize or recite information presented in a book. By *transfer*, we refer to an ability that goes beyond such learning: the ability to apply newly-acquired information to new exemplars or contexts. By *picture books*, we refer to books designed for pre-readers that contain pictures and may also contain text. We first present three developmental factors that may constrain learning and transfer from picture books. They have been selected because of their importance in supporting transfer of information across contexts, which is the focus of the studies we review here. We then provide a summary of studies investigating how features of picture books influence children's learning and transfer across a variety of educational domains by either reinforcing or working against the developmental processes presented. We conclude with ideas for new research and ways in which parents and educators can scaffold children's learning and transfer from picture books.

## Developmental factors influencing children's learning from picture books

Children's ability to transfer knowledge from picture books to the real world may be constrained by developments in their symbolic understanding, analogical reasoning, and their understanding of fantasy and reality. Although we discuss them separately, these areas of development are interwoven. As we will see, these developmental factors can be used to explain experimental findings on children's learning and transfer from picture books, as well as identify areas for future research.

### Symbolic development

One particular challenge that children may face when learning and applying real-world information from picture books is that of *symbolic insight* (DeLoache, 1991). That is, children need to be able to think flexibly about books as entities in themselves as well as symbolic sources of information about the world. For example, when reading an informational book about new animals such as South American cavies, children need to realize that they are reading a book with pages that can be flipped and pictures that tell a story about 2-dimensional cavies. They also need to recognize that the cavies on the page are intended to be representative of animals in the real world that have the same name (“cavies”) and features. Understanding that a picture in a book is an object that represents another entity is a symbolic task. This may not be a straightforward task for children especially since pictures in children's book can vary on the nature of their relation to the referent, that is, whether the picture represents real concrete (e.g., a cat) and abstract (e.g., letters and numbers) entities or imaginary entities (e.g., talking cats, talking pots, unicorns; Ganea and Canfield, 2015). Beyond the basic understanding that pictures are symbolic and stand for their referents, children will have to figure out what the nature of the referent is.

Young children often struggle with tasks that require symbolic reasoning. For example, 2-year-olds struggle to use information from videos and pictures of a room to help them find an object hidden in the real version of the room (Troseth and DeLoache, 1998). Despite the fact that these toddlers can easily point out and label the corresponding objects in the pictures and in the room, they do not transfer information from one to the other. Presumably this is because they think of the picture and the room each as a separate entity, and do not make the connection that the hidden object in the picture also represents a life-sized object hiding behind a pillow in the life-sized room. In addition, pictures in books are “impoverished” compared to information presented in real life because they provide only one visual perspective, lack depth cues like motion parallax and changing shadows, and may be low resolution. Simcock and DeLoache (2006) assert that perceptual differences between images in picture books and objects in the real world present a barrier to children's ability to use picture books symbolically, as a source of information about the world. This problem is not specific to young children's use of information from picture books, but from other symbolic media as well, such as videos (Anderson and Pempek, 2005; Barr, 2013). There is some evidence that transfer difficulties are similar across different media (books vs. videos; Brito et al., 2012), although there is also evidence of medium-specific differences in transfer (books vs. touchscreens; Strouse and Ganea, 2017). For the remainder of this review we will focus specifically on factors influencing young children's transfer from picture books.

Various features of picture books may differentially affect children's ability to treat the information symbolically. For example, pictures that more clearly represent the objects they depict may support children in recognizing the link between book depictions and the real world (Ganea et al., 2008; Ganea and Canfield, 2015). As such, unrealistic portrayals such as cartoonish images, fantastical settings, and depictions of animals with human characteristics may present particular challenges for children and will be reviewed below. Tactile features may pose a similar challenge, as they may highlight the book as an object, rather than as a symbol with information to be conveyed about the real world. These interactions between symbolic understanding and book features will be reviewed across various domains of learning below.

### Analogical reasoning

For successful transfer of complex information and concepts, children may need more than symbolic insight. To transfer basic information like the name of a novel animal from a picture book, children need to activate a representation of the animal in the book and remember details about its appearance to correctly apply the label to the real-world animal. To transfer more complex concepts, such as the ability for animals (in general) to use color camouflage to hide from predators, children must also recognize the abstract features of the depicted example and apply these to novel instances. Transferring conceptual information from one domain to another—in this case, from the picture book to the real world—requires children to recognize the abstract relational structure between the two domains (Gentner, 1989).

Children's ability to reason analogically depends somewhat on the difficulty of the task and their existing knowledge of the relations used in the analogy (Goswami, 1991). When they have experience in a domain, children as young as 1 or 2 years can use deep rather than surface features to solve analogical problems (e.g., Brown, 1990; Chen et al., 1997). However, when domain knowledge is limited, children without prior conceptual knowledge may be reliant on surface-level features to help them look for commonalities across analogical cases (Brown, 1989). One benefit of picture books as an educational resource is that they can provide children access to content that they would not experience in their day-to-day lives. However, this very feature of picture books may make analogical transfer especially difficult. For example, if children's understanding of color camouflage is tied to specific picture book illustrations (e.g., a frog) and surface features of that example (e.g., greenishness), they will likely fail to transfer the concept to other animals or contexts.

As with symbolic reasoning, various features of picture books may differentially affect children's ability to analogically transfer conceptual information in books. For example, given that perceptual similarity between transfer contexts facilitates analogical reasoning (Crisafi and Brown, 1986; Brown, 1989), children's transfer of new content from books with fantastical contexts and characters should be more impacted than transfer from books with realistic contexts and characters (Richert et al., 2009). If we expect children to learn and transfer novel content from picture books to a real-world context, stories that are more similar in surface structure to the real world would be easier for children to use a source of information about the world. Interactions between book features and analogical reasoning will be reviewed below.

### Reasoning about fantasy and reality

Children also have the challenge of determining which information in picture books should even be transferred. Anthropomorphism, or animals with attributes characteristic of humans, may be especially confusing when some information is meant to generalize and other information is meant to be true only in the story world. For example, if the cavies in a story talk and wear clothes, children must separate this anthropomorphization of cavies from factual information, inhibit transferring the unrealistic attributes, and selectively transfer only the factual information presented. Children's learning from picture books must be selective in that they have to separate what information is fictional versus what could be true in reality, which is generally referred to as the “reader's dilemma” (Potts et al., 1989; Gerrig and Prentice, 1991).

The process of keeping real-world knowledge separate from fictional or false information encountered in a story context may be especially difficult in early childhood because children between the ages of 3 and 8 are just beginning to differentiate fantasy and reality (Woolley and Cox, 2007). According to Woolley and Ghossainy (2013) young children are “naïve skeptics” when it comes to judging the reality status of fictional information. Instead of over-incorporating fantastical information into their real-world concepts, children err on the side of rejecting factual information presented. For example, 4- to 8-year-olds were more likely to state that an improbable event is impossible than to accept an impossible event as possible (Shtulman and Carey, 2007). A bias toward skepticism may impede transfer of educational information, as children may tend to not transfer details they are uncertain are “real.”

The ability to accurately distinguish reality and fantasy may also be related to children's representational development. Corriveau and Harris (2015) found that 3- to 4-year-olds accurately distinguished historical and fantastical characters in narratives at the same time that they started passing false belief and false signs tasks, suggesting that an understanding of representation (both mental and symbolic) may underlie the ability to distinguish fantasy from reality. Picture books, both in terms of their prose and illustrations, may be designed to represent reality or to represent make-believe. Corriveau and Harris (2015) argue that children may have difficulty deciding which of the two functions a particular story may fulfill. Thus, children's ability to separate fantasy from reality may depend both on their recognition that a story stands for *something* and their ability to judge what that something is (reality or pretend). In addition, children's own experiences and background knowledge may influence the aspects of stories they view as realistic versus fantastical (Corriveau et al., 2015).

Books with unrealistic content, such as impossible events or anthropomorphic depictions of animals, may present a challenge to children in separating which aspects of the book apply to the real world and which belong only in the book. Therefore, we again expect books with realistic content to be more supportive of learning transfer, especially when learning conceptual information such as scientific facts and concepts. Although these book features interact with the two other developmental factors discussed above—symbolic development and analogical reasoning—we also expect the developing ability to reason about what is real and what is fantastical to constrain or enable learning and transfer.

The following sections provide a review of how particular aspects of picture books (such as genre, pictorial realism, and the presence of manipulative features) interact with the three developmental factors we have proposed to influence children's transfer from picture books. We chose not to present this review as systematic or definitive, as research in many areas is in its early stages (see Table 1). Rather, we present information about how our identified developmental factors inform our understanding about children's learning from various book features and areas for further consideration in picture book research. We focus predominately on pre-readers who are listening to an adult read while they view the book's pictures.

**Table 1:** Summary of book features' impact on learning and transfer in each learning domain.

**Book feature**	**Word and letter learning**	**Biology**	**Physics**	**Problem solving**	**Moral learning**
**Pictorial Realism:** Transfer from photographs was easiest for infants, transfer from cartoons most difficult. With *symbolic development*, children get better at transferring from perceptually dissimilar depictions to real objects, so should get better at transferring from all kinds of pictures.	Ganea et al., 2008; Mareovich and Peralta, 2015	No studies	No studies	Simcock and DeLoache, 2006	No studies
**Manipulatives:** Features that distract from or obscure the basic correspondence between pictures and their referent appear to decrease learning in the word and letter learning and biological domains. With development of both *symbolic* and *analogical reasoning* skills, children should get better at overcoming this distraction.	Tare et al., 2010; Chiong and DeLoache, 2012	Tare et al., 2010	No studies	No studies	No studies
**Fantastical Contexts:** Young children have a tendency to err on the side of rejecting fantastical information, making transfer of information presented in fantastical contexts less likely. In addition, children need *analogical reasoning* skills to recognize contexts for application, which may be difficult because fantastical contexts necessarily differ from real-world contexts. Fantastical contexts appear to be most disruptive in the biological and problems solving domains and less disruptive in physical science, possibly because children are more willing to accept violations of reality in that domain.	Weisberg et al., 2015 (did not asses transfer)	Walker et al., 2014	Ganea et al., 2017	Richert et al., 2009; Richert and Smith, 2011	No studies
**Anthropomorphism:** Stories without anthropomorphism more clearly resemble reality, which may support the *symbolic insight* and *analogical reasoning* needed for children to recognize that information is relevant and should be transferred from one context to another. The ability to *distinguish fantasy from reality* may also support children in appropriately extracting information from anthropomorphic stories to be transferred.	No studies	Ganea et al., 2014; Waxman et al., 2014; Geerdts et al., 2015	No studies	No studies	Larsen et al., 2017
**Genre:** There is some evidence that the generic language used in information books could support children in identifying information that is intended to transfer, however the one study that directly compares learning and transfer of information from different genres found no effect. Development of *symbolic* and *analogical reasoning* skills may support children in identifying information to transfer regardless of the language used.	No studies	No studies	Venkadasalam and Ganea, 2017	No studies	No studies

## Domains of learning

Particular features of picture books, such as the specific content they incorporate, or the way in which the content is presented, may influence children's tendency to learn and transfer the educational content to real-world situations. Below we review studies that investigate some of these features, organized by the domain in which the educational content is presented. We have chosen this organization because particular features may be more influential in some learning domains than others. For example, visual features may be important when learning vocabulary, where children may be fairly successful at transfer on the basis of matching up perceptual features of objects. However, contextual information may be more important in science domains where transfer often takes place on a conceptual level. The domains we have chosen are primarily the domains in which the impact of picture book features on transfer of information presented in books have been studied. In each section, we address the book features that have been studied in that domain, interpreted with regard to our three developmental factors. Future work is needed to address how book features influence transfer in other domains such as math and the arts, as well as how additional book features impact transfer.

### Word and letter learning

Picture books expose children to rich language. For example, picture books contain a richer diversity of words (Montag et al., 2015) and a greater incidence of rare grammatical constructions (Cameron-Faulkner and Noble, 2013) than child-directed speech. In addition, caregivers use a larger number and wider variety of words during reading than other activities (Hoff-Ginsberg, 1991). It is not surprising, then, that joint reading has been associated with a variety of later language outcomes, including vocabulary growth and early literacy skills like letter knowledge (e.g., Bus et al., 1995). Here we are interested in particular features of books that may support the process of language learning from picture books on a less protracted scale—words and letters learned from individual reading sessions. We expect that symbolic understanding plays an especially important role in this domain, as transfer of a new word to a new context heavily depends on recognition of the labeled item in the book as representing objects in the real world to which the label also applies (Preissler and Carey, 2004; Ganea et al., 2008, 2009). Thus, features of books that make the link between depicted objects and real world referents clearer or easier to discern should support transfer, whereas features of books that make these links more difficult to recognize may make transfer more difficult. The book features that have been most studied in this domain include pictorial realism, manipulative features, and fantastical contexts.

#### Pictorial realism

Picture books vary in the degree to which their pictures represent reality, from photographs to illustrations to cartoonish line drawings. An image that is highly iconic, or visually very similar to its referent, may highlight the relation between the picture book image and real-world instances. As such, we might predict photographs to be the most supportive of children's transfer of knowledge from books to reality.

Newborn infants perceive and distinguish the dimensional nature of pictures from real objects. If presented with a complex object and a photograph of it, they clearly prefer the real object (Slater et al., 1984). However, when presented with photographs alone, 9-month-olds interact with them in ways similar to how they would interact with the real object they represent—by hitting, rubbing, and grasping the photographs (Pierroutsakos and DeLoache, 2003). Their behavior suggests they have not yet grasped the symbolic function of pictures.

As infants reach the middle of their second year, they begin to treat pictures referentially, by pointing and labeling the depicted objects (DeLoache et al., 1998). Research also indicates that in their second year of life children understand the representational status of pictures (Preissler and Carey, 2004; Ganea et al., 2009). Yet, children's transfer of novel words from picture books to the real world referent can be impacted by pictorial realism at these ages. Ganea et al. (2008) showed 15- and 18-month-olds picture books presenting both familiar and novel objects in the form of photographs, realistic color drawings that closely resembled the photographs, or color cartoons which were less detailed and more distorted in appearance. After being read the book by a researcher (told the names for the pictured objects), children of both ages were able to recognize the labeled object they had seen in the book regardless of the type of image. However, children who were read the cartoon book did not generalize to a picture of a new exemplar different in color. Eighteen-month-olds transferred the label to its physical real-word referent across all three conditions, but 15-month-olds did so only in the photograph and drawing conditions. Taken together, these findings suggest that transfer from the photographs was easiest for children, and transfer from cartoons the most difficult. With age, children get better at transferring from perceptually dissimilar depictions to real objects, although there is evidence that the iconicity of pictures continues to play a role in some picture transfer tasks even at 3 years of age (Callaghan, 2000; Mareovich and Peralta, 2015). The impact of iconicity on young children's learning from picture books has also been found with other measures, such as imitation (Simcock and DeLoache, 2006). Thus, at young ages, when children are first beginning to think symbolically, their understanding that pictures stand for real objects interacts with the type of depictions in books.

#### Manipulative features

The term “manipulative features” has been used to refer to features that are “designed to increase children's physical interaction with [a] book,” like lift-a-flap, scratch-and-sniff, and other three-dimensional add-ons (Tare et al., 2010, p. 396). These features may be entertaining for children, but research suggests they may not be optimal for learning. One reason they may not be optimal for learning is that they may draw attention away from links between the book and the real world. For young children who are still learning to use pictures in books as “standing for” real objects, this may distract from the insight necessary for transfer of learned information.

Using books designed to teach children animal names, Tare et al. (2010) tested the helping or hindering influence of manipulative features on 18- to 22-month-olds' learning and transfer of the animal names. Children were read a book by a researcher featuring 9 animals either using a commercially presented manipulative book (with flaps and pull tabs) or a scanned copy of the book (without manipulative features). At test, children who had seen a copy of the book without manipulatives correctly generalized a new animal name to new pictures and a replica of the animal. Children who read the book with manipulative features did not perform above chance. In another study, researchers compared 30- to 36-month-olds' learning of letters from a manipulative alphabet book with pulls, flaps and textures to a book without these features (Chiong and DeLoache, 2012). Children learned more letters from the simple alphabet book than the manipulative one. The authors argued that the salience of manipulative features may render them more like objects themselves and less like symbols that stand for other objects than their 2D counterparts. Children's difficulty transferring labels from manipulative books may therefore stem from a difficulty in “seeing past” the fancy features to realize that the content is representational, meaningful, and applicable to other contexts.

Another possibility is that children's mental effort is engaged with interaction with the features rather than attending to the content. For example, pulling a tab in an alphabet book to make a truck move does not help to emphasize the correspondence between the letter T and the first sound in the word “truck.” There is other evidence that features that require additional mental effort, like having multiple large pictures on each page, can result in cognitive overload, disrupting learning (Flack and Horst, 2017). Flack and Horst (2017) read 3- to 5-year-olds books with one or two regular-sized illustrations per page spread or one large image per spread. New objects in the pictures were labeled with new words during reading. At test, children were asked to identify the referent of the labels by pointing to the correct objects on a book page. Children were more successful when they had seen one illustration, regardless of size, indicating that two illustrations may have resulted in cognitive overload. The researchers did not assess transfer of learning. In a follow-up study, a hand gesture that directed children to the correct illustration supported learning from the book with two pictures per spread. In light of these effects of cognitive overload on children's learning, more research is needed to determine whether manipulatives are particularly disruptive of symbolic insight, whether they result in cognitive overload, or both.

Research shows that not all manipulative features are detrimental to children's learning. Multimedia researchers have argued that extra book features that engage children with the educational content of books (called “considerate,” Labbo and Kuhn, 2000) can support learning. A recent meta-analysis of studies involving electronic books with considerate enhancements like animated pictures, music, and sound effects were supportive of vocabulary learning for preschool and elementary children (Takacs et al., 2015). While we know of no similar results with manipulative features of print books, one study suggests that manipulatives designed to draw attention to the educational content, in this case the shape of letters, did not distract 3-year-olds from learning the letter names (Chiong and DeLoache, 2012).

For both word and letter learning, the manipulative features traditionally found in print books do not appear to facilitate learning and transfer, and in cases when the features are irrelevant to the book's educational content, may even interfere with it. Content-central manipulatives that highlight educational content, such as highlighting the visual shape of a letter—the crucial component for transferring the letter name to new instances of the letter—may hold promise in facilitating symbolic insight, and thus transfer. Research in this area will become especially crucial as the features available in digital books continue to expand.

#### Fantastical contexts

In picture books both fantastical and realistic, children may encounter new and unusual vocabulary. However, we might predict that realistic story contexts provide more cues to children that they can use to match story depictions and contexts with real-world situations. The similarity between the learning and transfer contexts can provide support for symbolic insight—recognizing the similarity between a symbol and its referent—as well as for analogical transfer. A recent intervention with low-income preschoolers investigated the effect of fantastical or realistic content on children's word learning (Weisberg et al., 2015). Children were presented with a set of realistic or fantastical commercial picture books and toys. The researchers measured children's comprehension of the vocabulary presented in the books and toys receptively and asked them to tell everything they knew about the tested word (e.g., “What are weeds?”). Across both conditions, children showed similar gains in identifying the tested objects. However, children in the fantastical condition were able to provide more information about the objects when given open-ended prompts. This study suggests that children learned more about the target objects in the fantastical contexts. Importantly, however, this study did not assess any type of transfer to the real world, and no distinction was made between fantastical and realistic information in explanations given by children. How fantasy may influence children's ability to transfer labels to new exemplars or real-world referents remains to be investigated. Consideration of the developmental factors we identified here—symbolic insight, analogical transfer, and reasoning about fantasy and reality—would lead one to predict that children will have more difficulty transferring labels from fantastical than realistic books to real-world referents.

#### Summary: picture books and word and letter learning

Picture books are a rich source of new language. Because infants and toddlers are just learning to use pictures symbolically to refer to other objects, features that support this insight rather than distract from it are most supportive. If the goal is to teach children new words or letters, it appears that books with realistic images are best, especially with the youngest children. If books with manipulative features are selected, they should draw attention to the educational content rather than distract from it. More research is needed to determine the influence of realistic versus fantastical contexts on children's transfer of new words they have learned to other contexts, as well as how these contexts interact with children's developing abilities to distinguish fantasy and reality. Future research could consider not only the variety of picture arrangements on a page (Flack and Horst, 2017) but also the type of backgrounds that pictures are displayed on and the type of object arrangements (whether an object is displayed with objects from the same category or a different category). An insightful analysis of the structure of children's books for children aged 0 and 3 was provided by Kummerling-Meibauer and Meibauer (2011) and future research could use it as a guideline to experimentally test what types of book structures are most inducive to young children's word and letter learning.

### Learning biological facts and concepts

Children's learning about non-human animals has been the focus of most studies of children's biology learning from picture books. Children are naturally interested in animals from a young age (DeLoache et al., 2011) and animals feature heavily in books designed for young children (Marriott, 2002). Thus, this domain for learning involves the largest amount of research on the impact of picture book features on transfer. A subset of studies has investigated biological concepts that apply to humans and non-human animals alike, including nutrition (Gripshover and Markman, 2013) and adaptation by natural selection (Kelemen et al., 2014). Another, reviewed here, focused on teaching children a novel biological causal relationship (Walker et al., 2014).

As is the case when learning the correspondence between words and letters and their referents, symbolic understanding can also play an important role in learning and transfer of biological facts and concepts. However, transfer of conceptual knowledge requires more than just symbolically matching a picture with its real-world referent; it often involves more complex reasoning about similarities between situations and selection of the correct details for transfer. Therefore analogical reasoning and discrimination between fantasy and reality should play a much more central role in young children's learning of biological information from picture books than it did for word and letter learning. The book features that have been studied in this domain include manipulative features, fantastical contexts, anthropomorphism, and genre.

#### Manipulative features

Concerns about the use of manipulative features in biology learning mirror those for word learning. When children are learning to symbolically link picture books and the real world, distracting features in books may disrupt that link. In one study with 27- to 39-month-olds, children were read either a pop-up book, a book with realistic images, or a book with drawings (Tare et al., 2010, Study 2). During book sharing, the experimenter told the child four facts about the dietary preferences of animals depicted in the books (e.g., chicks like to eat worms). Children who were read the pop-up book learned fewer facts from the book than children who were read the books without pop-up features. This study did not assess transfer of those facts to new contexts, but demonstrates that features that distract from or obscure the basic correspondence between pictures and their referent operate to decrease learning in the biological domain, as with word and letter learning.

#### Fantastical contexts

Although fantasy may be a much-loved and engaging genre, what do the violations of reality inherent to this genre mean for children's learning and transfer? Fantastical books may vary widely by mixing characters, settings, and events that vary in their realistic nature. Books with fantastical aspects could be an especially good choice for young children because they may engage children in imaginative thinking. Imaginative play may facilitate better causal reasoning (Walker and Gopnik, 2013), better deductive reasoning (Dias and Harris, 1988), and increased empathy for and understanding of others (Mar and Oatley, 2008). Parker and Lepper (1992) suggest that fantasy contexts may also be highly educational because they are engaging and motivating for children (see also Hopkins and Weisberg, 2017). However, fantastical contexts may make it more difficult for children to see links between books and reality, whether symbolically or analogically. Fantastical contexts may also make it more difficult for children to identify what information in books is real and should be transferred.

In a study of children's causal learning from realistic versus fantastical picture books, Walker et al. (2014) presented 3-, 4-, and 5-year-olds with one of two fictional picture books and tested their generalization of a fictional target biological causal relation: Popple Flowers cause hiccups when one sniffs them. The target relation was couched in either a realistic world (e.g., a boy climbs a tree) or a fantastical world (e.g., a boy has a conversation with a tree). In both books, the boy sniffs a popple flower and gets hiccups. Children were then asked to judge whether events in the story “could really happen” or “cannot really happen, and are just pretend.” Next, children were told by the experimenter that she smelled a Popple Flower earlier and asked whether they thought she did or did not get hiccups. When the fictional story world was more realistic, children were more likely to judge the target relation as something that “could really happen” and to predict that the experimenter got hiccups from smelling the flower herself. The tendency to transfer the target information from the more fantastical world decreased with age, as children's ability to distinguish fantasy and reality matured. This study indicates that when children are asked to transfer information from the story to a supposed real-world situation (a real person sniffing a Popple Flower) they rely on contextual information presented in the story to reason about whether the information should be transferred or not. In the case of the fantastical story, the context of the story world and the real world were less similar than in the case of the realistic story, thus decreasing the chance of analogical transfer. Also, as noted before, when children are uncertain about the fantastical status of information, they tend to be skeptical, erring on the side of caution when determining what is real. Fantastical contexts may cue children that information in the story is irrelevant to their situation and thus decrease their tendency to apply the information to realistic contexts.

#### Anthropomorphism

In an analysis of 1,064 modern picture books, Marriott (2002) concluded that picture books typically present the animal kingdom and its natural environment in an inaccurate and misleading manner, including a tendency toward anthropomorphism. Providing animals with habitats and traits that are realistic for humans may be an especially difficult type of fantasy for children to recognize, as these features may fit comfortably with their own personal experiences of the world. For example, it may seem plausible that animals would cry when sad or sleep with a blanket because those are part of children's everyday lives. Recent evidence demonstrates that children may struggle to distinguish between the anthropomorphic characteristics portrayed in stories and the real characteristics of animals. This struggle could influence the information that children transfer from stories to the real world.

In one study, Ganea et al. (2014) created two types of picture books about novel animals: one with factual language and another with anthropomorphic language. Both book types contained realistic images, and provided facts about each target animal. Across both book types, 3- to 5-year-olds who were read the books by a researcher learned the target facts presented in the picture books. Importantly, however, children who heard anthropomorphic stories about novel animals more often attributed anthropomorphic characteristics (e.g., feeling proud, having friends) to real animals in photographs than did those who heard the stories with no anthropomorphic language. Thus, children sometimes incorrectly transferred anthropomorphic attributes to real animals.

In a second study, Ganea et al. (2014) investigated the impact of anthropomorphic images on children's fact learning and tendency to anthropomorphize. They presented a new group of 3- and 5-year-old children with books about novel animals that contained either factual or anthropomorphic language. In this case, both book types included anthropomorphic illustrations (e.g., animals eating at a dinner table). Children in the full anthropomorphic condition (anthropomorphic images + language) answered fewer factual questions correctly than children in the anthropomorphic images only condition (with factual language). Children in the full anthropomorphic condition also attributed more anthropomorphic characteristics to real animals. These findings suggest anthropomorphic language may be particularly confusing for children.

Using storybooks with subtler forms of anthropomorphism, Geerdts et al. (2015) investigated the effects of anthropomorphism on 3- to 6-year-old children's learning about camouflage. In their anthropomorphic books, animals were portrayed with human-like faces and postures, but in their natural environment. Children read a picture book with either factual or anthropomorphized language, combined with either realistic or these subtler anthropomorphic pictures. In general, transfer was low—only a group of boys exposed to the book with the anthropomorphic pictures transferred information about camouflage to realistic situations at test, and there were no condition differences in the psychological properties children attributed to animals. The study had only 12 children per condition, so limited conclusions can be drawn about the lack of condition effects. Future research will need to address whether the style of anthropomorphic depictions has an impact on what children learn and transfer from stories.

Another recent study offers insights into how anthropomorphic depictions influence children's biological reasoning and learning. Waxman et al. (2014) told 5-year-olds a novel fact either about dogs or about humans (i.e., “Dogs/Humans have andro inside them.”). They then read children a few pages from an anthropomorphic book (Berenstain Bears) or a realistic book (an animal encyclopedia entry). After the realistic book children reasoned that bears had andro regardless of whether they had been told the fact about humans or dogs. After the anthropomorphic book, children reasoned that bears had andro only if they had been told the fact about humans. This study suggests that anthropomorphic portrayals may lead children to think of those animals as more human-like, and even a very brief exposure to depictions of animals in picture books (whether anthropomorphic or realistic) can influence the way they reason about non-human animals as having human traits.

Children's anthropocentric biases may also interact with the format of the books in which they encounter novel animals. We know that children from rural communities, who likely had more experience with nature, are less like to take an anthropocentric perspective than urban children (Waxman and Medin, 2007), perhaps because they have more first-hand experience that allows them to accurately identify anthropomorphic portrayals as fantastical. On the other hand, urban children who lack first-hand experience with a variety of animals may instead have anthropomorphic reasoning reinforced through other sources, such as media depictions (e.g., picture books) and conversations (Herrmann et al., 2010). These different anthropocentric biases may affect the extent to which children transfer information they encounter in a fantastical book about animals, with rural children less likely to transfer anthropomorphic information and urban children more so. Anthropomorphic depictions of animals in picture books may in turn increase children's tendency to consider animals as human-like, especially for children who have limited first-hand experience with other species. As researchers work to follow up the potentially positive roles that anthropomorphic characters may play, parents and teachers can work to dispel biological misconceptions by talking with their children about which characteristics are real and which are not (McCrindle and Odendaal, 1994; Marriott, 2002; Gebhard et al., 2003). Thus, supporting children's fantasy-reality distinction through discussion can support children who have not fully developed this ability to appropriately learn and apply information from books to the real world.

#### Genre

Children may also use book genre as a cue to determine whether information should be transferred to new contexts or is applicable only to story worlds. Children's books can be divided broadly into fiction (generally narratives) and non-fiction (informational, generally non-narrative) genres. Informational texts are realistic non-fiction books that are designed to convey information about the natural and social worlds (Duke, 2000). Informational books play an important role in classrooms; imagine learning organic chemistry or algebra without a textbook! Despite their prevalence in advanced classrooms, informational texts are rare in early childhood and early elementary classrooms (Pressley et al., 1996; Duke, 2000). Although sales in the informational book genre have grown in recent years, sales for children's fiction remain approximately four times higher (Milliot, 2015). The traditional absence of information books from early childhood contexts may be the result of a widely held assumption that narrative is the more effective genre for engaging children (Donovan and Smolkin, 2001; Duke et al., 2003; Mantzicopoulos and Patrick, 2011). However, a recent study found that preschoolers actually preferred information books over fictional ones, and teachers found the content more transferrable to real life (Kotaman and Tekin, 2017).

One hallmark of informational books is that they contain more generic language than narrative books (Gelman et al., 2012). Laboratory studies have shown that 3- and 4-year-olds are sensitive to differences in language and extend properties to larger categories when they hear generic language (Cimpian and Markman, 2008). Due to the differences in style of language used by the books, we might expect children to more readily transfer information from informational books. For example, a narrative book about cavies might contain the statement, “Dave the cavie eats fruit,” whereas an informational book might state, “Cavies eat fruit.” Based on Cimpian and Markman's (2008) findings we might predict that the generic nature of the second statement could act as a cue that all cavies eat fruit, rather than the specific cavie named Dave. However, it may be the case that children's generalization is robust to differences in genre and language specificity when the type of content applies at the category level (e.g., about diet). When mothers share picture books with children they provide both generic and specific language when offering natural facts about animals, suggesting generalizable facts are not consistently in generic language (Nyhout and O'Neill, 2014).

No studies have addressed learning biological information from non-narrative information versus narrative fiction specifically; however, one study compared two books where some of the language differed in specificity. Three- and four-year-olds were read one of two narrative picture books designed to teach the concept of color camouflage (Ganea et al., 2011). The factual book contained a combination of general statements about frogs interspersed with a narrative about a specific bird and frog called “the bird” and “the frog.” In the intentional book, the frog was named “Sammy” and generic statements about frogs were replaced with specific statements about Sammy. The intentional book also included statements anthropomorphizing the intentions of the animals, e.g., “Sammy tricked the bird.” Three- and four-year-olds successfully transferred information about camouflage to novel situations presented using photos of frogs and other animals regardless of which book they read. Four-year-olds also transferred to live animals in tanks. The study shows that children can transfer biological information from books to the real world when both types of language are used. Further research will be needed to establish whether generic language used in books provides a cue to children about transfer, as one may expect from other research, and whether other genre-related book features influence children's learning.

#### Summary: picture books and biology learning

Differences in book features appear to have significant effects on children's ability to extract and transfer biological information to the real world. Fantastical contexts used in stories may cue children that information presented in books is not transferrable to real-world contexts. Because children tend to err on the side of caution when reasoning about what events could really happen, children may fail to apply accurate biological information presented in fantastical stories, dismissing it as unrealistic. In contrast, anthropomorphic details in stories appear to push children's reasoning in the opposite direction—influencing children to reason about animals as similar to humans and potentially motivating them to accept inaccurate biological information about animals. This may be mediated by experience; children without extensive experience with animals may use their own personal (human) experience to help them distinguish what is realistic. Adults may help to dispel misconceptions about animals by talking with children about the characteristics portrayed in stories. In either case, realistic books may more readily support analogical transfer by portraying contexts similar to the real world and characteristics that are appropriate for transfer.

Finally, book genre has the potential to support transfer via its use of stylistic features such as language and image type. More research is needed to determine the extent to which the specificity of language used or other genre-related features support children's acquisition of biological information from picture books. Contexts that more clearly resemble reality may support both the symbolic insight needed for learning in transfer in children's early acquisition of biological facts from books (e.g., chicks eat worms) and the analogical reasoning needed for later acquisition of scientific concepts (e.g., camouflage).

### Physics

The task of learning physics concepts is similar to that of learning biological concepts in many ways. First, information may require conceptual abstraction beyond lining up surface features—e.g., both natural selection and centrifugal force apply in situations that vary greatly in context. Thus, picture book features that are based on visual similarity (like pictorial realism) may be less important for supporting transfer than features that support insight into analogical contexts. However, the necessary mismatches between the fantastical details in stories and real-world contexts may make it more difficult for children to recognize similarities between the contexts, thus disrupting analogical transfer. Second, realistic and unrealistic information about both biology and physics is often mixed together in children's stories, making fantasy-reality distinctions particularly difficult. For example, in *The Magic School Bus and the Electric Field Trip*, children are taught about electricity through a narrative in which the school bus shrinks to the size of an electron—violating certain laws of physics while intending to teach others. The necessary mismatches between the fantastical details in stories and real-world contexts may make it more difficult for children to recognize similarities between the contexts, thus disrupting analogical transfer. Despite their similarities, however, there is reason to expect that children will treat information about biology and physics differently. Sobel and Weisberg (2014) found that 4-year-olds who constructed a story were more likely to include events involving physical violations (e.g., walking through a wall) than biological ones (e.g., aging backwards), indicating that children found reality-violating physical events and contexts more acceptable than reality-violating biological events in their stories.

Two very recent studies indicate that books appear to be good tools for teaching children transferrable concepts about physics. Ganea et al. (2017) found that 6- and 7-year-olds with misconceptions about balance showed improved understanding of balance on a real-world task regardless of whether they were read a realistic or fantastical book about balancing a see-saw. The majority of children maintained this improvement at a follow-up visit after a 1-week delay. In another study, 4- and 5-year-olds learned and transferred information about gravity and falling objects equally well from an informational or narrative picture book read to them by a researcher (Venkadasalam and Ganea, 2017). From the sparse evidence available, transfer of physical science concepts does not appear to be easily disrupted by manipulations of fantastical context or genre as in other domains, although more research, using a broader range of concepts, is needed. In addition, both studies reviewed here involved children learning accurate real-world physical information from books. Future research on fantastical contexts should address whether children are able to discriminate accurate physics information from violations of reality (e.g., shrinking busses) and appropriately apply the real but not the fantastical information to real-world situations.

### Problem solving

Problem solving occurs when one wants to achieve a goal and no obvious solution occurs to the problem-solver (Mayer and Wittrock, 1996). The problem solver accesses their own knowledge and skills to develop a solution. When the problem solved is different from problems encountered previously, this involves a process of transfer. As with all problems of transfer, the problem solver must recognize similarities between what was originally learned and the new context—in this case, similar features of problems. The child must also recognize the solution in the story as a representation of a problem solution that is potentially relevant to events beyond the book context. Symbolic reasoning may help children recognize that information is symbolic and transferrable, and analogical reasoning skills may help children identify potentially relevant contexts for transfer. Thus, we may expect children's skills in these areas to be especially relevant when transferring problem solutions from stories to the real world.

An interesting feature of problem-solution transfer is that is can often occur after a substantial delay. A child may not encounter a relevant real-world problem until days, weeks, or even months after reading the story. The child must recall and recognize the abstract similarities between the story problem and the problem they face that goes beyond the surface features of the two problems. For example, a story character may retrieve a ball stuck in a rafter using a broom. The child may later use a similar strategy to retrieve a ball stuck in a tree using a hockey stick.

As we discuss in more detail below, children's ability to distinguish fantasy and reality may also influence their transfer of problem solutions. Problem solutions present in fantastical stories can be relevant to the real world, and children with a better grasp of possibility may be better able to apply solutions from fantasy to the real world. Children who approach fantastical events with skepticism are unlikely to transfer solutions from these types of stories.

#### Pictorial realism

In problem solving tasks that can be solved with some reliance on visual similarity, pictorial realism can impact young children's transfer. Books that incorporate pictures that are more similar to real objects, like photographs, help children align book objects with their real-world referents, and transfer skills they have learned from a book. Simcock and DeLoache (2006) showed 18-, 24-, and 30-month-olds a picture book which portrayed the assembly of a ball, jar, and stick into rattle. After a delay, they were given real versions of the objects and asked tested on whether they assembled the pieces into a rattle. Children at all ages assembled the rattle when they had read a book with color photographs of the objects. Children in the two older age groups transferred the solution from color line drawings, and only children in the oldest group transferred the solution from the book with pencil drawings. This study shows that the pictorial realism of the pictures in the book influenced children's transfer of the rattle assembly, and that this book feature interacts with development. When realistic photos are used, even 13-month-olds can use information presented in a picture book to make inductive inferences about non-obvious properties of real objects and attempt to elicit those properties through particular actions that were depicted in the book (Keates et al., 2014; see also Khu et al., 2014 for a study using the same task).

#### Fantastical contexts

Simcock and DeLoache's (2006) task required transfer of a solution in which the learning and transfer contexts were highly visually matched. However, as with transfer of scientific concepts, transfer of problem solutions often requires considering deep features rather than surface-level characteristics. This requires skill in analogical reasoning. There are also important differences between transfer of science concepts and problem solutions. In the case of biology and physics, children are tasked with separating realistic from unrealistic information and only transferring that which is applicable to the real world. In the case of biology, this appears to often be difficult for children, as they are not good at distinguishing the two and tend to err on the side of rejecting anything that may seem unrealistic. However, for those who can distinguish appropriately, a lack of realism may act as a useful cue that particular information should not be transferred.

In problem solving, however, the ability to distinguish between realistic and unrealistic information may be less important because solutions to fantastical problems are often applicable to real-world situations if deep features are considered. Even children who can appropriately distinguish fantastical portrayals may struggle to apply problem solutions optimally because their skepticism toward applying fantastical information may lead them to dismiss solutions presented in fantastical contexts even when the problem solution would apply to real-world problems.

In one study, 3- to 6-year-olds were read two “social interaction” stories (joining a friend group and taking another's perspective) and two “physical solution” stories (wrapping and stacking) featuring either human or fantastical characters (Richert et al., 2009). Children more readily transferred solutions to real-world social and physical problems from a story with real characters than one with fantastical characters.

Similarly, Richert and Smith (2011) compared 3- to 5-year-old children's ability to transfer solutions for novel problem types presented in full-length, commercial picture books when read by a researcher. Children were presented with a point-of-view problem, in which the solution was for the character to hide from an individual by standing behind him, and a pulling problem, in which the solution was to attach a suction cup attached to a rope to move an object. Again, children were more likely to transfer the solution to the real world when the problems had been presented in a realistic version of the picture book than a fantastical version.

Similar to the pattern seen in the biological domain, fantastical contexts appear to make transferring problem solutions to real-world situations more difficult for children. In problem solution tasks, children need to identify analogical similarities between a problem presented in a book and a problem faced in the lab. Skill in fantasy-reality discrimination may support children in realizing that problem solutions in fantastical contexts may apply to real world problems. In support of this interpretation, Richert and Schlesinger (2016) found that 3- to 6-year-old children with a better understanding of the fantasy-reality distinction were better able to learn and transfer problem solutions from video stories when fantastical elements were present and relevant to the solution being presented. Fantastical elements that were incidental appeared to distract children and interfere with transfer. More research is needed to identify other features of books that influence children's transfer of problem solving strategies.

### Moral learning

Many popular children's characters have encountered a bully, lied, or had bad dreams. Adults may choose these books hoping they will teach children information they can use in their own daily experiences. However, adults should not assume that pre-readers readily extract the moral messages intended by authors. Even as late as third grade, children have difficulty identifying the moral themes of oral stories when asked to explicitly describe them (Narvaez et al., 1998). These researchers report that children often choose responses that have superficial characteristics in common with the story rather than appropriate thematic responses.

As with science learning and problem solving, children cannot rely on surface-level features to extract moral themes. As such, we might expect analogical reasoning and fantasy-reality distinction to play important roles in learning moral messages. As with problem solving, although morals presented in unrealistic contexts may be applicable to real-world situations, even children with the ability to distinguish fantasy and reality may tend not to transfer moral lessons.

In addition to the challenges discussed in other domains, learning thematic messages from books may be an additionally difficult task because children must learn to connect together the relations and events that occur across multiple story events. According to van den Broek et al. (2005), this ability emerges at the end of a developmental sequence: first, young children hearing stories begin by making connections between physical events that occur close together in the story. Then, they progress to making connections between more distant and abstract events, followed by clustering events by theme. Once children are able to make these connections, they can use them to extract a story's moral or lesson, an ability requiring analogical reasoning. This developmental sequence unfolds gradually throughout early childhood, possibly making the transfer of moral messages to the real world one of the most difficult domains for learning from picture books. As a result, we might expect transferring morals to be more easily disrupted by book features, but unfortunately, little research is available in this area.

Larsen et al. (2017) tested whether animal characters with human characteristics were better for teaching transferrable morals than human characters using books intended to encourage sharing. Four- to six-year-olds were read either a commercial picture book about an anthropomorphized raccoon who learns that sharing makes her feel good or a version of the book in which the raccoon characters were replaced with humans. Both before and after reading children were given stickers and the opportunity to share some of the stickers with another child who would not have the opportunity to receive any. Children who had read the story with the human characters shared significantly more stickers after than before the book sharing. Those who read the book about anthropomorphized raccoon shared significantly fewer stickers after than before book sharing. Of interest is the finding that children who judged anthropomorphized animals as more human-like (on a categorization task using stimuli unrelated to the main picture books in the study) were those who were most likely to share after hearing the anthropomorphized animal story, suggesting that a lack of identification with the characters could have contributed to lack of transfer of the moral theme. Also, perceived similiarity with the story characters may make it more likely for the child to grasp the intent of the story and apply it to their own lives. Stories are created with the intention to communicate something and to adults the communicative intention behind a story may be straightforward, however children may need more support to be able to identify the story's intended message.

There is additional evidence that human characters may be supportive for helping children identify and extract story themes. Another study, which did not involve a transfer task, found that 4- and 5-year-olds were more likely to identify the theme of a story they were read (ask permission to join a game) if it featured human characters than if they were read the same story with rabbit characters (Kotaman and Balci, 2017). The children who were read the human story also scored better on general story comprehension.

The available research suggests that characters that are, or are perceived as, similar to the child may enhance the extraction of story morals and their transfer to real-world situations. As with other domains, transfer of moral themes depends on children's ability to see the similarity between the situation in books and real-world situations. Realistic characters may be one way of supporting this connection. In addition, characters and contexts that differ greatly from real-world contexts may lead children to question which information in stories is realistic and should be transferred.

## Concluding comments

Adults and children regularly engage in joint reading with a variety of goals. In this review, we have focused on the use of books to teach children transferrable information about words, letters, science, problem solutions, and moral lessons. Through this review, a few important themes have emerged.

First, children's learning from a given picture book appears to be the result of an interaction between the particular features of the book, the type of information to be learned, and constraints on children's development in the areas we have outlined. As we have seen, certain features (e.g., fantasy) may be more disruptive in some domains (i.e., problem solving and moral lessons) than others (i.e., word and physics learning). Children's age and therefore developmental stage also affects what and whether they learn. For example, pictorial realism and manipulative features may be especially disruptive for younger children in word and letter learning where transfer can occur based on aligning surface-level features such as shape and color. In this domain the development of symbolic understanding may help in instances when mismatches between pictures and reality or distacting features interfere with transfer between book and real contexts. This same interaction between book features and development may not be as important in domains like problem solving and morality where children need to understand and transfer deeper features across situations rather than rely on surface-level features. As another example, fantastical contexts may be more detrimental for a child who has not yet worked out how to reliably separate the possible from the impossible because he/she is unlikely to accurately select transferrable information from fantastical stories. However, when children achieve a better grasp of this distinction, fantastical stories may not present as much of a barrier to learning in domains where fantasy serves as a good cue for lack of transferability.

Second, there is still much that we do not know about which features support learning from books. Each feature has been tested only a handful of times in a handful of contexts. While some features, such as realistic portrayals of animals, may be optimal for teaching biology, the reverse may be true for encouraging empathy for animals and nature. For example, children often use anthropomorphic reasoning to explain why trees and other elements of nature should be protected (Gebhard et al., 2003). Different patterns of anthropomorhims effects on children's learning may also emerge at different ages (Geerdts, 2016; Severson and Lemm, 2016). Table 1 displays the domains and book features that have been discussed and allows for identification of areas which have not been studied.

Finally, the most supportive thing adults can do to help children learn, even more than selecting high-quality books, is to have conversations with them during reading. Adults reading books with manipulative features, be they traditional or electronic, may support children by focusing less on the hands-on features and drawing attention back to content-related talk. When it comes to choosing information for transfer, adults may use generic language to signal to children that particular information is true across contexts (Gelman et al., 2012). More generally, effective methods for supporting children in transferring conceptual information from one story context to another are to talk with children about the underlying structure of the story (Brown et al., 1986), ask them to teach it to someone else (Crisafi and Brown, 1986), or prompt them to explain (Walker and Lombrozo, 2017). Other dialogic reading techniques such as asking children questions, helping them extract themes, and having them help tell the story across repeated readings may also be supportive of transfer. Parents and teachers may use our review to help select potentially educational books, but reading and talking together can make any book-reading session educational and pleasurable.

## Author contributions

All authors developed the structure and content of the manuscript. GS and AN drafted the manuscript. All authors provided edits and feedback.

### Conflict of interest statement

The authors declare that the research was conducted in the absence of any commercial or financial relationships that could be construed as a potential conflict of interest.
